# Low sulfated heparan sulfate mimetic differentially affects repair in immune‐mediated and toxin‐induced experimental models of demyelination

**DOI:** 10.1002/glia.24363

**Published:** 2023-03-21

**Authors:** Susan L. Lindsay, George A. McCanney, Jiangshan Zhan, Miriam Scheld, Rebecca Sherrard Smith, Carl S. Goodyear, Edwin A. Yates, Markus Kipp, Jeremy E. Turnbull, Susan C. Barnett

**Affiliations:** ^1^ School of Infection and Immunity University of Glasgow 120 University Place Glasgow G12 8TA UK; ^2^ Institute of Anatomy University of Rostock Gertrudenstrasse 9 18057 Rostock Germany; ^3^ Institute of Neuroanatomy, Faculty of Medicine RWTH Aachen University 52074 Aachen Germany; ^4^ Institute of Systems, Molecules and Integrative Biology University of Liverpool Liverpool L69 7ZB UK; ^5^ Centre for Glycosciences Keele University Keele ST5 5BG UK

**Keywords:** CNS repair, cuprizone, EAE, heparan sulfate, multiple sclerosis, myelination

## Abstract

There is an urgent need for therapies that target the multicellular pathology of central nervous system (CNS) disease. Modified, nonanticoagulant heparins mimic the heparan sulfate glycan family and are known regulators of multiple cellular processes. In vitro studies have demonstrated that low sulfated modified heparin mimetics (LS‐mHeps) drive repair after CNS demyelination. Herein, we test LS‐mHep7 (an in vitro lead compound) in experimental autoimmune encephalomyelitis (EAE) and cuprizone‐induced demyelination. In EAE, LS‐mHep7 treatment resulted in faster recovery and rapidly reduced inflammation which was accompanied by restoration of animal weight. LS‐mHep7 treatment had no effect on remyelination or on OLIG2 positive oligodendrocyte numbers within the corpus callosum in the cuprizone model. Further in vitro investigation confirmed that LS‐mHep7 likely mediates its pro‐repair effect in the EAE model by sequestering inflammatory cytokines, such as CCL5 which are upregulated during immune‐mediated inflammatory attacks. These data support the future clinical translation of this next generation modified heparin as a treatment for CNS diseases with active immune system involvement.

## Main Points

- Modified heparan sulphate mimetics are next generation therapeutics for CNS repair.
- Using two animal models of demyelination a low sulphated heparan sulphate mimetic was found to promote repair of immune‐mediated not toxin‐induced demyelinating disease.

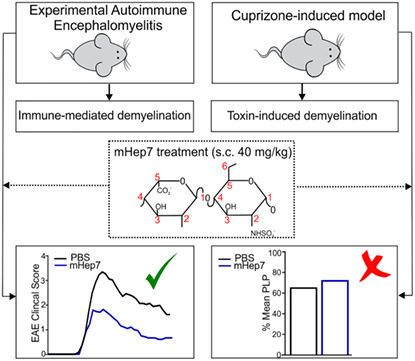

## 1 INTRODUCTION

Numerous pathologies of the central nervous system (CNS) result in demyelination and neuronal damage. There is a pressing need for the identification of a therapy that drives repair at a multicellular level. Key master regulators of numerous cellular functions are the heparan sulfate (HS) glycan family. HS interacts with many proteins via “codes” of variant sulfated polysaccharide structures (Bishop et al., 2007) and can be modulated by exogenous heparin species such as, unmodified heparin, shorter heparin oligosaccharides, and various nonanticoagulant derivatives of different sizes (Casu et al., 2010). Heparins are currently highly effective and safe anticoagulants in the clinic, which has paved the way for “next generation” modified nonanticoagulant heparins for new indications.

HS are anionic, sulfated, linear polysaccharides attached to protein cores forming a complex type of glycoproteins. They form a subgroup of the glycosaminoglycan (GAG) family and are essential components of the extracellular matrix (ECM), basement membrane and cell surface (Sarrazin et al., 2011). HS can interact and regulate many proteins, known as HS‐binding proteins including chemokines, cytokines, growth factors, proteases, adhesion proteins, and lipid binding proteins (Xu et al., 2018). They exert their biological effects through their modulation of protein activity, although the molecular details are not yet fully elucidated (Sarrazin et al., 2011; Xu et al., 2018), however, variations in HS sulfation patterns or epimerization (D‐GlcA to IdoA) results in structural diversity, which is considered to underpin their numerous biological functions. HS have been demonstrated to have fundamental roles in CNS physiology (Fitch & Silver, 2008; Hartmann & Maurer, 2001; Smith et al., 2015). They interact with several growth factors/cytokines known to modulate signaling cascades that could benefit remyelination. These include the binding of fibroblast growth factor 2 (FGF2), known to influence OPC differentiation (Bogler et al., 1990), heparin‐binding EGF‐like growth factor, which affects myelination (Nakagawa et al., 1998), as well as Semaphorin 3A and 3F, known regulators of OPC migration (Williams et al., 2007). They also influence myelination through synapse formation (Smith et al., 2015). In addition, HS is important during inflammation as shown by their effect on the activities of leukocyte migration across the blood–brain barrier (BBB), which could contribute to the formation of inflammatory CNS lesions (Farrugia et al., 2018; Floris et al., 2003; Simon Davis & Parish, 2013). Their dysregulation is implicated in neurodegenerative diseases as well as numerous other pathologies, including cardiovascular disease, cancer, inflammation, metabolic diseases, and viral infections (Bishop et al., 2007; Roy et al., 2014).

Nonanticoagulant, structurally modified heparin mimetics have previously been generated (Yates et al., 1996) and can influence the function of many processes within the CNS (Duckworth et al., 2015; McCanney et al., 2019; Patey et al., 2006; Schworer et al., 2013; Tyler et al., 2015). These mimetics are semisynthetic since they are synthesized by the chemical modification of heparin, for example, the selective desulfation of single or multiple sulfate groups on the repeating sugars that make up heparin. Modified heparins have been shown to be an effective treatment in animal models of Alzheimer's disease and are anticancer drug candidates (Duckworth et al., 2015, Patey et al., 2006, Schworer et al., 2013, Tyler et al., 2015). Moreover, we have recently demonstrated that HS mimetics (mHeps) can be used to modulate endogenous mechanisms for potential therapeutic benefit after CNS injury (McCanney et al., 2019). These studies, using embryonic spinal cord derived myelinating cultures (MC) (Nash et al., 2011; Sorensen et al., 2008; Thomson et al., 2008), have shown that highly sulfated mHeps have adverse effects on the biological properties of CNS neural cells (Higginson et al., 2012; McCanney et al., 2019; O'Neill et al., 2017). In comparison, low sulfated (LS)‐mHeps and in particular LS‐mHep7, reverse the induction of astrogliosis (Higginson et al., 2012) and, promote myelination of demyelinated axons and extension of nerve processes in vitro (McCanney et al., 2019). We showed that after in vitro injury, soluble protein factors were released that inhibited CNS repair which were abrogated by the addition of LS‐mHeps. In support of this concept, we used an unbiased proteomics approach by applying TMT‐LC/MS to LS‐mHep7 affinity purified conditioned media (CM) and identified multiple heparin binding factors, including amyloid beta A4, and several other chemokines and cytokines (McCanney et al., 2019). Amyloid beta A4 was further validated as an inhibitor of myelination (McCanney et al., 2019) and has been by other shown to inhibit OPC differentiation (Horiuchi et al., 2012) in vitro. Combined, these findings suggest that, after injury, protein factors are released or up‐regulated that inhibit CNS repair and LS‐mHeps act to bind these factors, sequestering their negative effect, in turn promoting repair (McCanney et al., 2019).

The complexity of CNS injury will not be overcome by targeting a single cell or factor and the advantages of LS‐mHeps is their multimodal action, which target the multifactorial nature of CNS disease processes and provide a novel route for the development of a disease‐modifying therapeutic. To begin to harness this potential, in vitro studies have identified a lead compound, termed LS‐mHep7 which, by the removal of *O*‐sulfate groups from C2 and C6 positions, creates a predominantly mono‐*N*‐sulfated compound that lacks anticoagulant activity (Patey et al., 2006). Herein, we have tested the efficacy of LS‐mHep7, in, experimental autoimmune encephalomyelitis (EAE) and cuprizone‐induced demyelination, two in vivo models used widely in preclinical screens of potential therapeutics for MS. Both these complimentary in vivo techniques culminate in oligodendrocyte degeneration and the disruption of myelination, with hallmarks of demyelinating disease such as those observed in MS, albeit via distinct mechanisms. Notably, however, the EAE model is commonly used to investigate immune‐mediated demyelinating disease (resulting in consistently demyelinated inflammatory plaques in diverse brain regions, and among the lumbar spinal tissue) whilst the cuprizone model is noninflammatory (causing oligodendrocyte degeneration, apoptosis and demyelination in distinct brain regions, such as the corpus callosum) (Goldberg et al., 2013; Hesse et al., 2010; Schmidt et al., 2013). Using these complementary models, we show for the first time that treatment with the low sulfated HS mimetic LS‐mHep7 leads to improved animal outcomes, via the prevention of inflammation leading to axonal preservation and remyelination in the EAE model.

## 2 MATERIALS AND METHODS

### 2.1 Low sulfated modified heparan sulfate 7

Low sulfated modified heparin 7 was produced semisynthetically by selective chemical desulfation of heparin, essentially as previously described (Yates et al., 1996). The compound had the predominant disaccharide repeating structure IdoA‐GlcNS with corresponding chemical desulfation at both the 2‐O and 6‐O positions.

### 2.2 Experimental autoimmune encephalomyelitis model

Twenty‐seven female C57Bl/6J mice were purchased from Harlan Laboratories (Loughborough, UK). All mice were housed under a 12 h light/dark cycle with ad libitum access to food and water in pathogen‐free conditions. All experimental procedures were performed in accordance with the UK Animals (Scientific Procedures) Act 1986. All applicable international, national, and/or institutional guidelines for the care and use of animals were followed. The research protocol was approved by the Ethical Committee for Animal Experimentation in the University of Glasgow, UK. EAE was induced in female mice (7–8 weeks of age, weighing 18.5 ± 1.5 g) by subcutaneous (s.c.) injection at one site at the tail base with an emulsion (100 μL total) containing 150 μg myelin oligodendrocyte glycoprotein peptide spanning amino acids 33‐55(NH2‐MEVGWYRSPFSRVVHLYRNGK‐COOH, 98% purity) (SynPeptide, China) in complete Freund's adjuvant (Sigma‐Aldrich) supplemented with 300 μg Mycobacterium tuberculosis (strain H37RA; Difco). Mice were injected intraperitoneally with 200 ng pertussis toxin (Sigma‐Aldrich) in 100 μL of phosphate buffer saline solution (PBS, pH 7.6) immediately, and 48 h after the immunization. After induction, mice body weight were monitored daily and EAE clinical scores using the following ascending flaccid paralysis clinical scoring system: 0 = no clinical signs, 0.5 = partially limp tail, 1 = paralyzed tail; 2 = impaired gait; 2.5 = hindlimb paresis; inability to hindlimb weight bear; 3 = partial hindlimb paralysis; 3.5 = one hindlimb paralyzed; 4 = total hind leg paralysis; 5 = any sign of forelimb paralysis; 6 = moribund/dead. When mice achieved a score of 1 on the EAE clinical scale (loss of tail tone) they were randomly assigned to either LS‐mHep7 or PBS control group (Figure S1a). Mice received 100 μL s.c. injections of 40 mg/kg LS‐mHep or PBS every‐other‐day until the end of the experiment. Terminal endpoints were considered as a weight loss of more than 20% of animal starting body weight and/or any signs of forelimb paralysis (score of 5).

### 2.3 Acute cuprizone model

Twenty‐four C57BL/6 male mice (8–10 weeks, 17–27 g) were kept under standard laboratory conditions (12‐h light/12‐h dark cycle, controlled temperature 22 ± 2°C, and 50% ± 10% humidity) with access to food and water ad libitum. All experimental procedures were approved by the Review Board for the Care of Animal Subjects of the district government (Regierung Oberbayern; reference number 55.2‐154‐2532‐73‐15; Germany) and reported according to the ARRIVE (Animal Research: Reporting of In Vivo Experiments) guidelines (Kilkenny et al., 2010). Cuprizone [bis–cyclohexanone‐oxaldihydrazone] intoxication was performed by the addition of 0.25% cuprizone in the standard rodent chow for 5 weeks. Control animals received a diet of standard rodent chow for the entire duration of the study. After 5 weeks, normal chow was provided to the animals to allow for endogenous remyelination. During this remyelination period, eight animals were treated with LS‐mHep7 40 mg/kg via s.c. and eight vehicle control animals received PBS every‐other‐day for 14 days (Figure S1b). Thereafter, experiments were sacrificed for immunohistochemical studies.

### 2.4 Tissue processing

EAE mice under deep anesthesia were transcardially perfused with 4% paraformaldehyde. Spinal cords were removed and postfixed by immersion in the same fixative containing 30% sucrose at 4°C for 24 h, then washed and left in PBS containing 30% sucrose at 4°C until being frozen. Five millimeter spinal cord tissue blocks encompassing regions L2 until S1 were frozen on dry ice in OCT and consecutive 5 μm thick sections were cut, mounted onto SuperFrost™ slides (ThermoFisher, UK) and numbered in order of cutting. Cuprizone mice were anesthetized with ketamine (100 mg/kg i.p.) and xylazine (10 mg/kg i.p.), and then transcardially perfused with ice‐cold PBS followed by a 3.7% formaldehyde solution (pH 7.4). Brains were postfixed overnight in a 3.7% formaldehyde solution at 4°C, dissected, and embedded in paraffin. Coronal sections (5 μm) at the levels 255–265 of the mouse brain atlas (Sidman et al., 1971) were prepared onto SuperFrost™ coated slides (ThermoFisher, UK) using a microtome.

### 2.5 EAE immunofluorescence staining

Inflammatory demyelination was measured by staining for myelin basic protein (MBP) with anti‐MBP antibody (1:200, BioRad) and axonal loss was assessed using anti‐SMI‐31 (1:1000, Bio‐Legend). The inflammatory activity was analyzed using anti–CD45 (1:100, R&D systems) anti‐CD4. We used anti‐glial fibrillary acidic protein (GFAP, 1:500, Dako) for the detection of astrocytes. All primary antibodies were detected with appropriate conjugated secondary antibody (1:500, Thermofisher, UK) according to established standard protocols. Classic hematoxylin and eosin staining was used to analyze inflammatory activity. In brief, sections were stained with hematoxylin for 5 min, rinsed with distilled water then incubated in 1% acid ethanol for 10 s and rinsed sufficiently. Eosin was then applied for 5 min and rinsed sufficiently. Sections were dehydrated in gradient ethanol solutions and transparentized with xylene before being embedded with DPX mounting media.

### 2.6 Acute cuprizone model immunohistochemistry

Established protocols were used for immunohistochemistry (Slowik et al., 2015). In brief, sections were rehydrated and, if necessary, antigens were unmasked with citrate buffer (pH 6.0) and microwave heating. After washing in PBS, sections were incubated overnight (4°C) with the primary antibodies diluted in blocking solution (serum of species in which the secondary antibody was produced). The following primary antibodies were used: anti‐PLP (1:5000, Serotec, MCA839G; Puchheim, Germany), anti‐MBP (1:500, Abcam, ab7349; Cambridge, UK), and anti‐OLIG2 (Millipore). The next day, slides were incubated with biotinylated secondary antibodies (both 1:200, Vector Labs; anti‐mouse, BA9200; anti‐rat, BA9400) for 1 h and then with peroxidase‐coupled avidin‐biotin complex (ABC kit; Vector Laboratories, Peterborough, UK) and treated with 3,3′‐diaminobenzidine as a peroxidase substrate. Stained sections were digitalized using a Nikon ECLIPSE E200 microscope (Nikon Instrument) equipped with a DS‐2Mv camera.

### 2.7 Image acquisition and quantification

Quantification of antibody labelling in EAE sections was performed in the same area in sequential sections of the same spinal cord regions between groups. Two images per section, with a minimum of two sections per animal were quantified (Figure S2). Images were captured at 20 × magnification with an Olympus BX51 microscope using Ocular software and analyzed using Fiji (Image J). The captured images were quantified in a blinded manner. After image acquisition the white matter region of interest (ROI) in each image was manually outlined using ImageJ. Binary thresholding measurements were made of both the total ROI and of any positive staining and both converted into the number of black pixels. The staining intensity was then presented as the mean of % threshold/area. For quantification of SMI or MBP loss, using Image J, a grid containing 130 crosses was applied across each white matter ROI on ×40 magnification images. Each cross falling on a region of abnormal pattern of staining was counted, and the number of regions divided by the total number of crosses covering the entire ROI and expressed as percentage of abnormal staining/tissue area per field of view. Quantification of H&E‐stained inflammatory infiltration was made by counting the number of nuclei present within the same spinal cord white matter ROI in at least two spinal cord sections/animal, imaged on a Nikon ECLIPSE E200 microscope (Nikon Instrument) equipped with a DS‐2Mv camera at 40 × magnification. In the cuprizone model, stained sections (2 per animal) were evaluated by densitometry of the staining intensity using ImageJ and automated thresholding, as described for EAE. The corpus callosum ROI was manually outlined using either the open‐source program ViewPoint Online (PreciPoint, Freising, Germany) or ImageJ (MBF Bioscience Williston, ND, USA). The staining intensity was then presented as the mean of % area/field of view. For analysis of OLIG2 numbers, a 1 mm^2^ box was placed over the corpus callosum region on 10 × magnification images. Individual cell counts were made using ROI analyze particles in Image J and presented as counts/mm^2^. Positive staining densitometries, measurement of analyze particles and total ROI densitometries were calculated by an evaluator blinded to the treatment groups.

### 2.8 Rt‐PCR

Gene expression studies were performed on corpus callosum tissue isolated from five healthy and five animals fed 0.25% cuprizone in the standard rodent chow for 3 weeks, followed by 17 days on normal chow, and on spinal cord tissue isolated from four healthy and three EAE mice on day 17 post EAE induction. Total RNA was extracted using peqGOLD TriFast (VWR), and RNA concentration and purity were measured with the NanoDrop 1000 device (Thermo Scientific). cDNA synthesis was performed using moloney murine leukemia virus reserve transcriptase kit and random hexanucleotide primers (Thermo Fisher Scientific). cDNA levels were then analyzed by RT‐rtPCR using AceQ® qPCR SYBR Green Master Mix (Vazyme). The expression levels were calculated relative to the reference gene coding for hypoxanthin‐guanin‐phosphoribosyl‐transferase using the ΔΔCt method. Primer sequences and individual annealing temperatures are shown in Table 1.

**TABLE 1:** Details the sequences and individual annealing temperatures of the primers used in this investigation.

Gene	Sequence	Product size	Annealing temperature
CCL5	s GCTGCTTTGCCTACCTCTCC	104 bp	65°C
	as TCGAGTGACAAACACGACTGC		
HPRT	s TCAGTCAACGGGGGACATAAA	142 bp	65°C
	as GGGGCTGTACTGCTTAACCAG		

### 2.9 In vitro de/myelinating assays

Myelinating rat embryonic CNS cultures were grown using methods previously described (Lindsay et al., 2013; McCanney et al., 2019). Briefly, the upper 5 to 6 mm section of cervical embryonic day 15.5 spinal cord was removed from SD rats, cleared of meninges, chopped finely, and trypsin/collagenase dissociated. After soyabean trypsin inhibitor/DNase treatment, cells were triturated and spun at 800 rpm for 5 min. Cells were resuspended in plating medium (50% DMEM, 25% horse serum, 25% HBSS), 2 mM L‐glutamine (Invitrogen, Paisley, UK) and plated at 150,000 cells onto a monolayer of neurosphere derived astrocytes (Smith et al., 2022; Sorensen et al., 2008), left to attach for 3 h then fed with differentiation media (DM; DMEM; 4500 mg/mL glucose), 10 ng/mL biotin, 0.5% hormone mixture (1 mg/mL apotransferrin, 20 mM putrescine, 4 μM progesterone, 6 μM selenium) formulation based on N2 mix of (Bottenstein & Sato, 1979) 50 nM hydrocortisone and 10 μg/mL insulin (all reagents from Sigma; Invitrogen, Paisley, UK). Cultures were maintained by replacing half of the medium three times a week. After 12 days, insulin was removed from the DM and cultures treated with 100 ng/mL CCL5 (R&D systems, P13501) or CCL5 in combination with LS‐mHep7 (100 ng/mL) every other day until day 28. Alternatively, for demyelinating assays (DeMy), on day 24 cultures were treated with anti‐MOG (Z2 hybridoma), IgG2a (Piddlesden et al., 1993) and 100 μg/mL rabbit serum complement (Millipore), as previously described (Elliott et al., 2012; McCanney et al., 2019). CM was collected from cultures for further analysis either 24 h (DeMy CM D0) or 5 days after demyelination (DeMy CM D5). Alternatively, after demyelination, cultures were treated twice with DM or DM supplemented with CCL5 (100 ng/mL), or CCL5 (100 ng/mL) and LS‐mHep7 (100 ng/mL) or non‐demyelinated cultures were left as controls. Cultures were maintained for a further 5 days before processed for immunohistochemistry.

### 2.10 Immunohistochemistry and quantification of in vitro cultures

Cultures were fixed, stained, and quantified for levels of myelination using standard methods (McCanney et al., 2019). Briefly, primary antibodies, anti‐SMI‐31 (1:1000, Biolegend), and anti‐PLP (AA3 Hybridoma, 1:100) were diluted in blocking buffer, and incubated for 1 h. After washing, cultures were incubated with appropriate secondary antibodies for 45 mins and mounted in Vectashield (Vector Laboratories, UK). For every biological replicate, 10 random images per coverslip (three coverslips per condition) were taken for quantitative analysis at 10× magnification (neurite density and myelination) using an Olympus BX51 fluorescent microscope with a Retiga R6 and Ocular software (QImaging). Quantification of myelination was carried out using CellProfiler Image Analysis software (Broad Institute) (Lindner et al., 2015). CellProfiler uses pattern recognition software to distinguish between linear myelinated internodes and oligodendrocyte cell bodies. In this manner, we track the coexpression of myelin sheaths (PLP) and axons (SMI31), which ignores oligodendrocytes that do not contact axons, and therefore allows us to calculate the percentage of myelinated fibers. All experiments were carried out at least three times. All CellProfiler pipelines were developed in‐house and are available at https://github.com/muecs/cp.

### 2.11 LS‐mHep7 column pull‐down assay

CM collected from demyelinated cultures as described previously, were affinity purified using a HiTrap NHS‐activated HP column onto which LS‐mHep7 was bound, as previously described (McCanney et al., 2019). Briefly, a HiTrap NHS‐activated HP column was loaded with 10 mg/mL of LS‐mHep7 for 2 h at 15°C. Unbound LS‐mHep7 was washed with ddH2O and soluble amine added to react with any remaining *N*‐hydroxysuccinimide groups for 2 h at 15°C. Finally, the column was washed with PBS. For the LS‐mHep7 binding protein pull‐down, 6 mL of CM was filtered through a 0.22 μm membrane then run down the column. Unbound material was washed, and LS‐mHep7 bound proteins were eluted. The eluted heparin binding proteins were desalted and concentrated using an Amicon Ultra‐15 centrifugal device.

### 2.12 Rat Cytokine Array Panel A Proteome Profiler

The LS‐mHep7 eluate from control and demyelinated cultures was analyzed using a Proteome Profiler Rat Cytokine Array, Panel A (R&D Systems, ARY008). This 29‐plex array captures: CCL3, CXCL10, IL‐4, CCL5, CXC3CL1, IL‐6, CCL20, GM‐CSF, IL‐10, CNTF, ICAM‐1, IL‐13, CXCL1, IFN‐γ, IL‐17, CXCL2, IL‐1α, L‐Selectin, CXCL3, IL‐1β, TIMP‐1, CXCL5, IL‐1ra, TNF‐α, CXCL7, IL‐2, VEGF, CXCL9, IL‐3. Briefly, captured proteins were detected using biotinylated detection antibodies, following which, chemiluminescence enabled visualization, producing a signal relative to amount of protein bound. Each eluate was separately analyzed on individual arrays, however the blots were visualized and captured together using the Azure C500 (Azure Biosystems), to facilitate direct interarray comparisons. Quantification was carried out using the densitometry analysis tool on the Total Lab Quant Software.

### 2.13 CCL5 ELISA

To quantify the concentration of specific proteins in CM and LS‐mHep7 eluates a C–C Motif Chemokine Ligand 5 (CCL5, R&D systems, MMR00) ELISAs was performed as per manufacturer's description. Plates were read at 492 nm on a Tecan Sunrise™ automated microplate reader (Tecan Group Ltd, Switzerland) using the provided Magellan™ software.

### 2.14 Statistical analysis

Parametric data are presented as means ± SEM. Differences between groups were statistically tested using the software package GraphPad Prism 6 (GraphPad Software Inc., San Diego, CA, USA). The applied statistical procedures are provided in the figure legends. The number of experimental replicates (*n*) are shown as individual data points on the graph. *p* values < .05 were considered statistically significant. The following symbols are used to indicate the level of significance: *p < .05, **p < .01, ***p < .001.

## 3 RESULTS

### 3.1 LS‐mHep7 ameliorates experimental autoimmune encephalomyelitis

**FIGURE 1:**
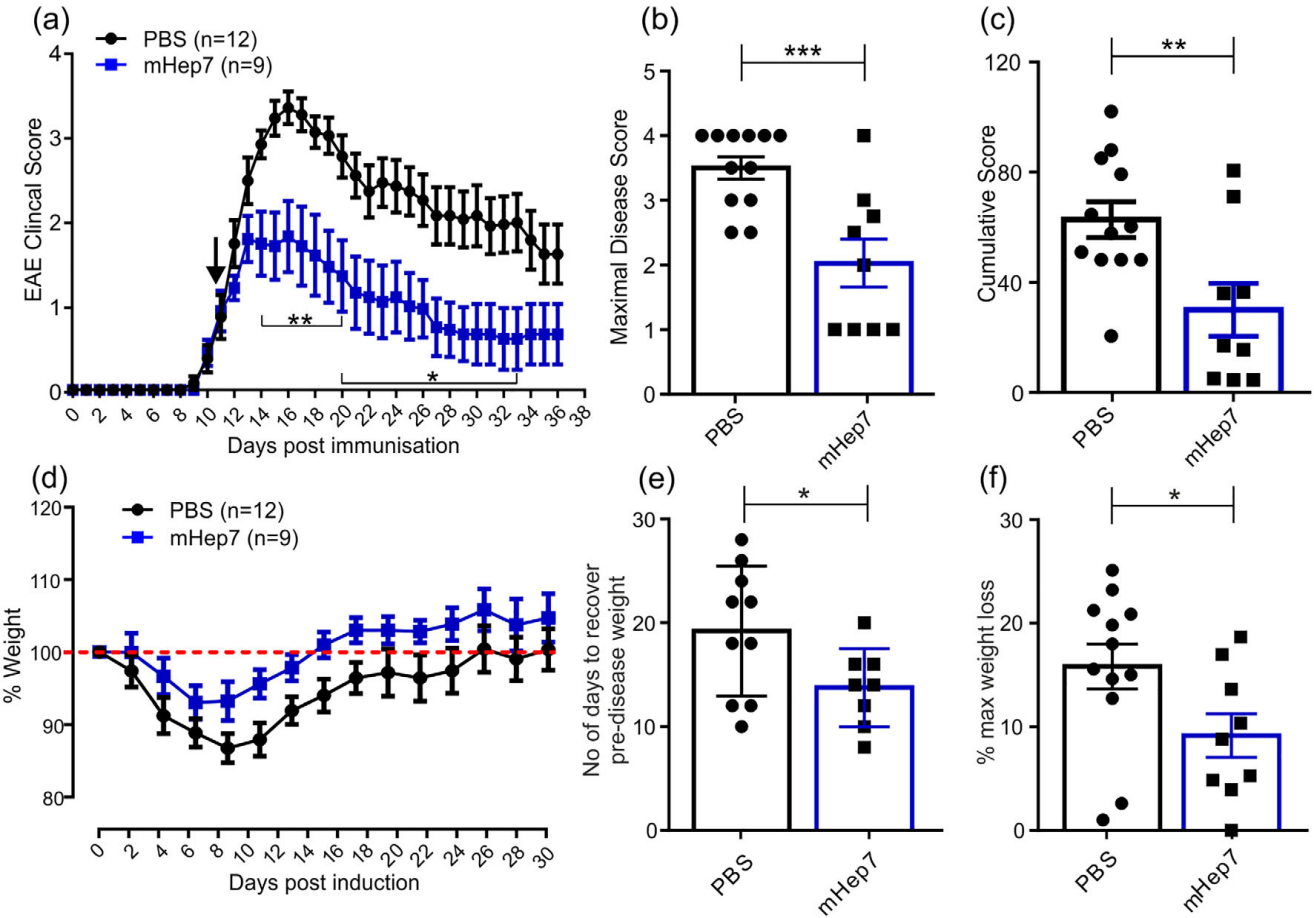
Effect of LS‐mHep7 on EAE disease course. (a) EAE was monitored over 36 days and s.c. injections of LS‐mHep7 (40 mg/kg) were administered every‐other‐day from when animals lost tail tone (score 1). LS‐mHep7 treatment caused a decrease in clinical severity compared with control animals. (b) Maximal disease scores and (c) Cumulative disease scores were both significantly reduced in LS‐mHep7 treated animals compared with PBS treated animals. (d) LS‐mHep7 treatment prevented the typical animal weight loss and promoted a faster recovery to predisease weight (red dashed line). (e) Animals required significantly less time to recover their predisease weight. (f) The percentage maximum weight loss of each animal was also significantly less in LS‐mHep7 treated animals compared with PBS injected control animals (Individual dots represent the experimental animal number, Student's unpaired two tailed *t* test, *p < .05, **p < .01, ***p < .001).

We initially investigated a therapeutic treatment regime of LS‐mHep7 to ameliorate EAE disease progression. In brief, when animals showed any signs of tail tone loss (average score of 1) they were randomly assigned to either a PBS or LS‐mHep7 treatment group. Mice received 40 mg/kg of LS‐mHep7 via s.c. injection every other day for 28 days. Notably, treatment with LS‐mHep7 suppressed disease progression compared with PBS injected animals (Figure 1a). This was associated with a significant reduction in the maximal disease scores (Figure 1b) and cumulative disease scores (Figure 1c). An alternative quantitative marker of disease progression and severity, which generally occurs just prior to disease onset, is weight loss. Interestingly, evaluation of the LS‐mHep7 treatment regime demonstrated that it lowered the level of animal body weight loss that is associated with normal EAE disease onset (Figure 1d). In addition, the number of days for animals to recover their predisease body weight (Figure 1e) and the maximal animal weight loss (Figure 1f) was significantly less in LS‐mHep7 treated animals compared with PBS injected animals. Combined these data suggest that treatment with LS‐mHep7 had a positive therapeutic benefit resulting in faster recovery and overall health.

### 3.2 Treatment with LS‐mHep7 reduces inflammation and CNS pathology in EAE

**FIGURE 2:**
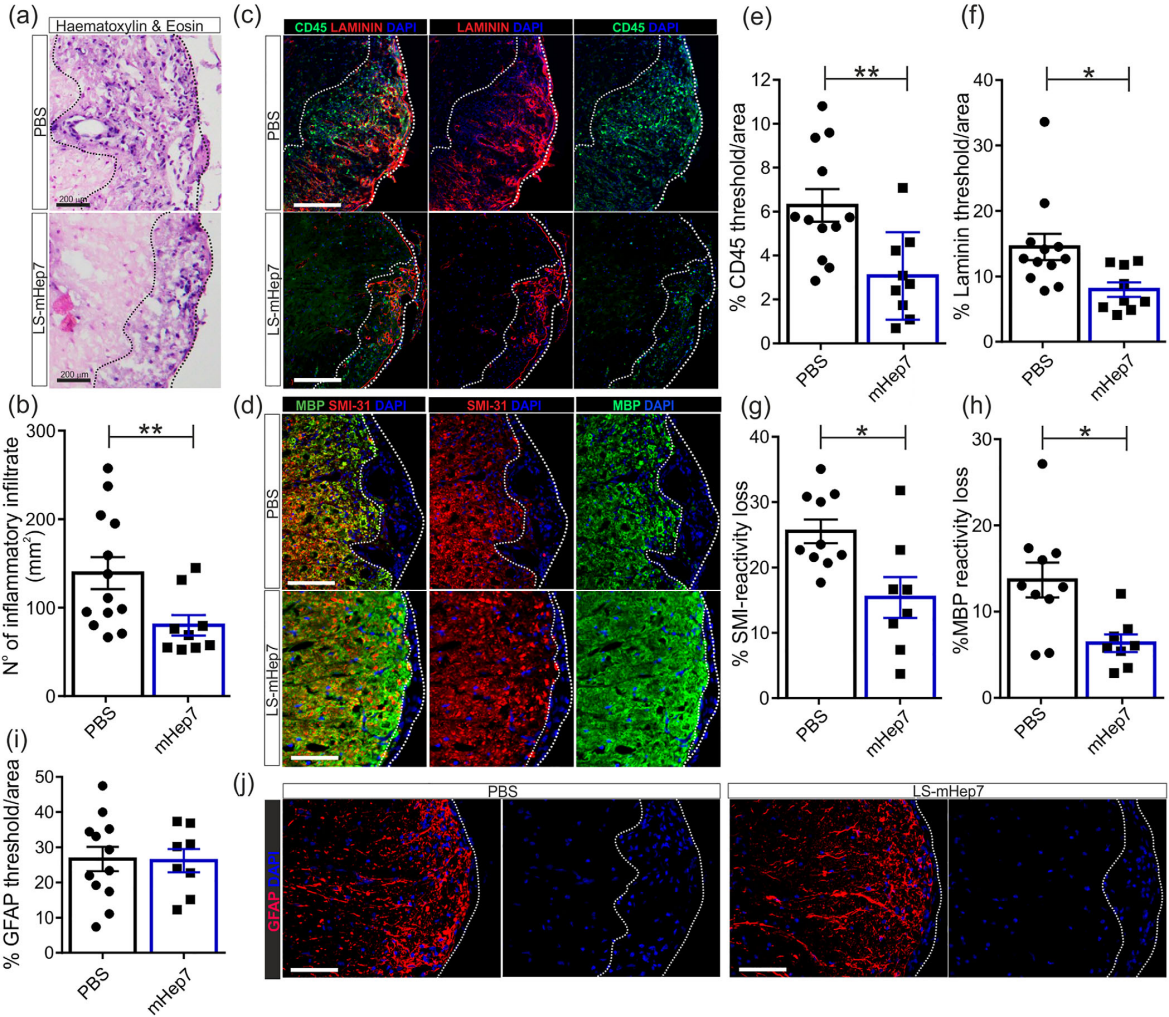
Effect of LS‐mHep7 on inflammatory infiltration, axonal and myelin loss, astrocyte reactivity within EAE spinal cord tissue. (a) Representative images of hematoxylin & eosin‐stained thoracolumbar spinal cord sections of animals that were injected with 40 mg/kg LS‐mHep7 every‐other‐day from loss of tail tone compared with PBS injected animals at the end of disease course. (b) Quantification of the number of nuclei present in inflammatory plaques showed that LS‐mHep7 treated animals had significantly less cellular infiltration. (c) Representative images of anti‐CD45 (shown in green), anti‐Laminin (shown in red) and DAPI (shown in blue) stained thoracolumbar spinal cord sections and (d) anti‐MBP (shown in green), anti‐SMI‐31 (shown in red) and DAPI (shown in blue). (e) Quantification of the % CD45 expression determined that LS‐mHep7 injected animals had significantly less leukocytes within the inflammatory plaques compared with PBS injected animals. (f) Quantification of the % laminin expression showed that LS‐mHep7 injected animals had significantly less expression compared with PBS injected animals. (g) Quantification of the % of abnormal axons by measuring the number of SMI‐31 lacking regions in inflammatory plaques, revealed that LS‐mHep7 injected animals had significantly less axonal loss compared with PBS injected animals. (h) Quantification of the % myelin loss by measuring the number of MBP lacking regions in inflammatory plaques, determined that LS‐mHep7 injected animals had significantly less abnormal MBP pathology compared with PBS injected animals. (i) Quantification of the % GFAP expression determined that LS‐mHep7 injected animals had similar levels of astrocyte reactivity. (j) Representative images of anti‐GFAP (shown in red) and DAPI (shown in blue) stained thoracolumbar spinal cord sections. (Individual dots represent the experimental animal number, *p < .05, **p < .01, Student's unpaired two tailed *t* test).

To interrogate the impact of LS‐mHep7 on the pathological consequences of EAE, we examined lumbar spinal cord tissue at the experimental endpoint. This revealed, as expected, that mice who received PBS had severe inflammation in the spinal cord. In comparison, treatment with LS‐mHep7 substantially reduced signs of inflammation (Figure 2). Quantitative analysis of spinal cord inflammatory cell infiltration showed that LS‐mHep7 treated mice had fewer cells within inflammatory plaques (Figure 2a,b). To further characterize the level of inflammatory infiltration, sections were stained with the pan leukocyte marker CD45, confirming the significant reduction in LS‐mHep7 treated animals (Figure 2c,e). To complement the inflammatory cell infiltrate we also evaluated the expression of laminin (a major component of the endothelial cell basement membrane of the BBB), as its expression is upregulated in inflammatory demyelinated lesions during EAE (Roscoe et al., 2009). Notably, we observed that LS‐mHep7 treatment resulted in a significant decrease in laminin expression (Figure 2c,f). To determine whether the decreased inflammation was associated with reduced CNS pathology, we measured both axonal and myelin loss (Figure 2d). Assessment of axonal densities, via SMI‐31 reactivity within inflammatory lesions, revealed that LS‐mHep7 treatment led to significantly less damage (Figure 2d,g). Correspondingly, there was also a reduction in myelin loss, as assessed by MBP immunoreactivity (Figure 2d,h). To determine whether LS‐mHep7 treatment modulated astrocyte reactivity, measurements of GFAP reactivity within spinal cord sections were made (Figure 2i,j). There were no differences in the level of GFAP immunoreactivity in treated animals compared with control animals.

### 3.3 LS‐mHep7 quickly prevents disease accumulation

**FIGURE 3:**
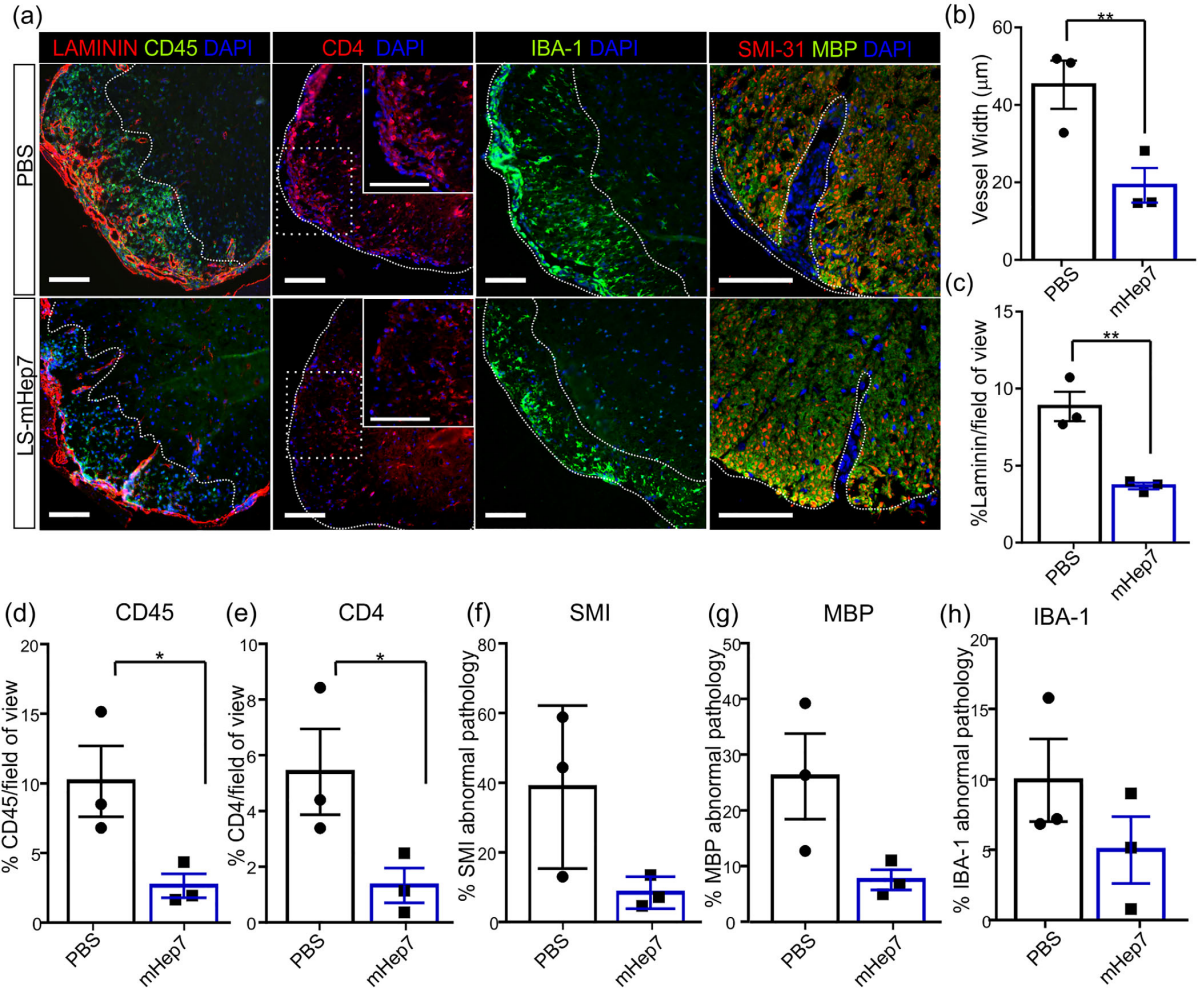
Effect of LS‐mHep7 on inflammatory infiltration, axonal, and myelin pathology 5 days posttreatment. (a) Representative images of anti‐CD45 (shown in green), anti‐Laminin (shown in red) & DAPI (shown in blue); and anti‐CD4 (shown in red) & DAPI (shown in blue), higher magnification inset boxes of area delineated by dashed white square; and anti‐IBA1 (shown in green) & DAPI (shown in blue); and anti‐MBP (shown in green), anti‐SMI‐31 (shown in red) & DAPI (shown in blue), stained thoracolumbar spinal cord sections of animals that were injected with only two doses of 40 mg/kg LS‐mHep7 every‐other‐day over 5 days from loss of tail tone compared with PBS injected animals. Scale bars represent 100 μm. (b) Disruption of the blood brain barrier was assessed by measuring blood vessel width which was significantly reduced in LS‐mHep7 treated mice compared with controls. (c) Correspondingly, there were reduced levels of laminin expression in LS‐mHep7 treated animals compared with PBS control. (d) Quantification of the % CD45 expression and (e) the % CD4 expression determined that LS‐mHep7 injected animals had significantly less leukocytes within the inflammatory plaques in white matter compared with PBS injected animals. (f) Quantification of the % of abnormal axons by measuring the number of SMI‐31 lacking/abnormal regions in inflammatory plaques, revealed no significant difference between LS‐mHep7 injected animals and PBS injected animals. (g) Quantification of the % myelin loss by measuring the number of MBP lacking regions in inflammatory plaques, revealed no significant difference between LS‐mHep7 injected animals and PBS injected animals. (h) Quantification of % IBA‐1 expression showed no difference in microglia activation in LS‐mHep7 treated animals compared with PBS controls. (Individual dots represent the experimental animal number, *p < .05, **p < .01, Student's unpaired two tailed *t* test).

Next, we investigated the impact of LS‐mHep7 after only two doses (5 days posttreatment) by examining lumbar spinal cord tissue. We found that like that found at experimental end point, there was a significant reduction in inflammatory immune cells as both CD45 and CD4 expression were significantly reduced in LS‐mHep7 treated animals versus PBS (Figure 3a,d,e). This corresponded to a reduction in the disruption of BBB as assessed by measurement of both blood vessel width (Figure 3a,b) and the extent of laminin expression (Figure 3a,c). Although not significantly different at this early time point, there was also a trend toward reduced levels of axonal pathology (Figure 3a,f) and myelin loss (Figure 3a,g). There were no significant differences in IBA‐1 staining in LS‐mHep7 animals (Figure 3a,h). These data suggest that LS‐mHep7 has a quick effect in dampening down the inflammatory cascade, preventing the accumulation of immune cell recruitment after only two doses.

### 3.4 LS‐mHep7 has no effect on remyelination in the acute cuprizone model

**FIGURE 4:**
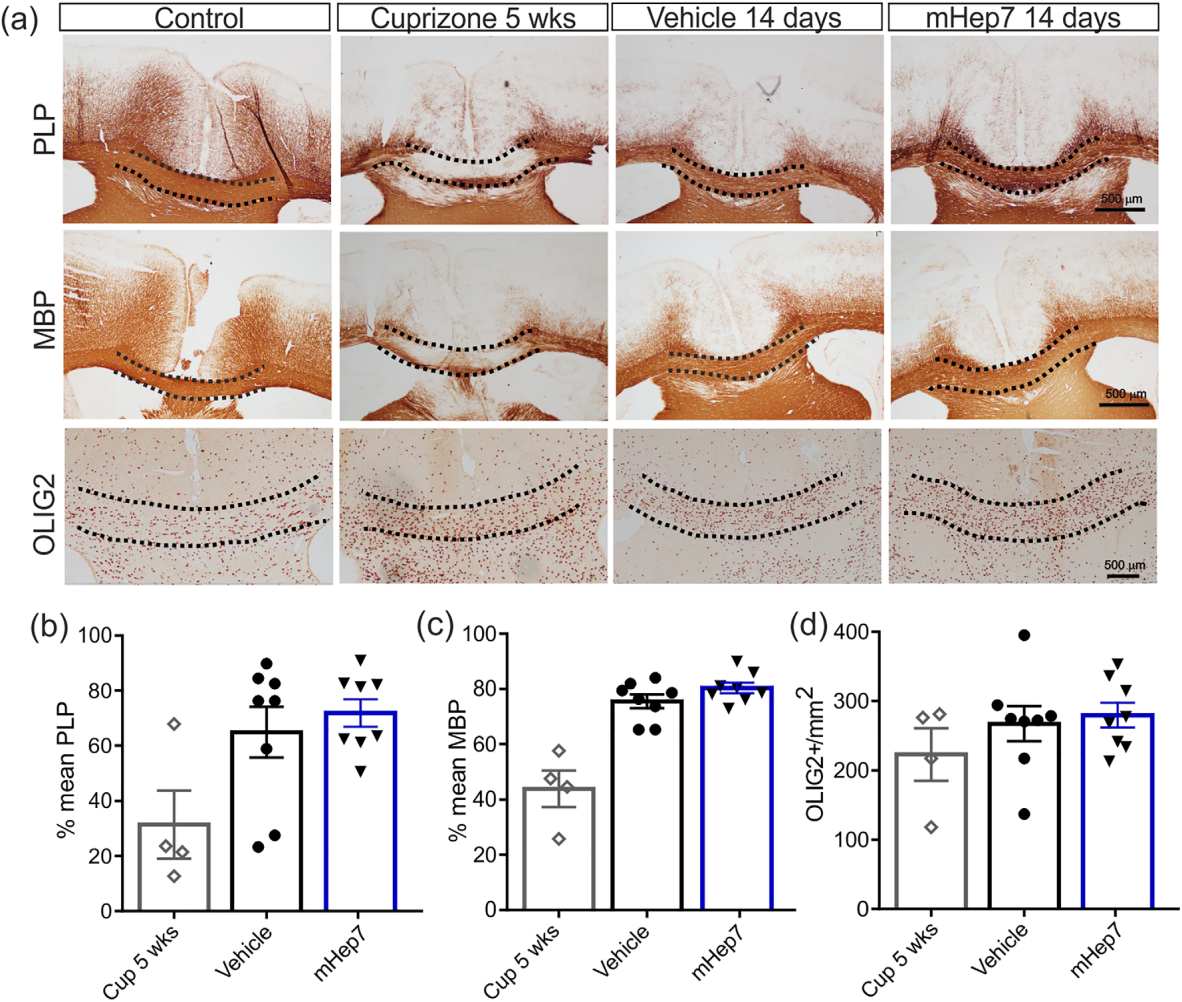
Effect of LS‐mHep7 on PLP, MBP, OLIG2 expression in the acute cuprizone model of demyelination. (a) Representative images of anti‐PLP, anti‐MBP, and anti‐OLIG2 staining within anatomical brain region 215 of a control animal, or after being fed cuprizone diet (0.25%) for 5 weeks (Cup 5 weeks) or after treatment with PBS vehicle or LS‐mHep7 40 mg/kg via subcutaneous injections (s.c.) for 14 days post cuprizone diet removal. Dashed black line demarcates the corpus callosum (CC) region. (b) Thresholding measurements of the CC region using Image J algorithm “huang” showed no differences in the levels of PLP or (c) MBP remyelination after LS‐mHep7 treatment compared with the vehicle control group. (d) There was no difference in the OLIG2 counts after LS‐mHep7 treatment compared with control animals. (Individual dots represent the experimental animal number, *p < .05, One‐way ANOVA, Tukey's multiple post‐comparison test).

Given the ability of LS‐mHep7 to suppress inflammatory‐driven CNS pathology and the associated faster recovery in EAE, it was also important to understand whether LS‐mHep7 also had the capacity to accelerate myelin repair. To this end, we used the toxin induced de‐ and re‐myelination cuprizone model. Mice were intoxicated with cuprizone for 5 weeks, then transferred to a normal chow diet and treated with either PBS, or LS‐mHep7 (40 mg/kg) via s.c. injections for 2 weeks. Evaluation of both MBP and PLP within the corpus callosum of animals fed cuprizone chow for 5 weeks revealed severe loss of both marker expression correlating with demyelination (Figure 4a). LS‐mHep7 was unable to modulate the level of either PLP or MBP recovery post‐cuprizone intoxication (Figure 4b,c). There were also no differences in OLIG2^+^ oligodendrocytes numbers in LS‐mHep7‐ compared with vehicle‐treated animals (Figure 4a,d). These data demonstrate that LS‐mHep7 does not have the ability to modulate remyelination in this model at the 2‐week time point evaluated.

### 3.5 Role of CCL5 in LS‐mHep7 mechanism‐of‐action after demyelination

**FIGURE 5:**
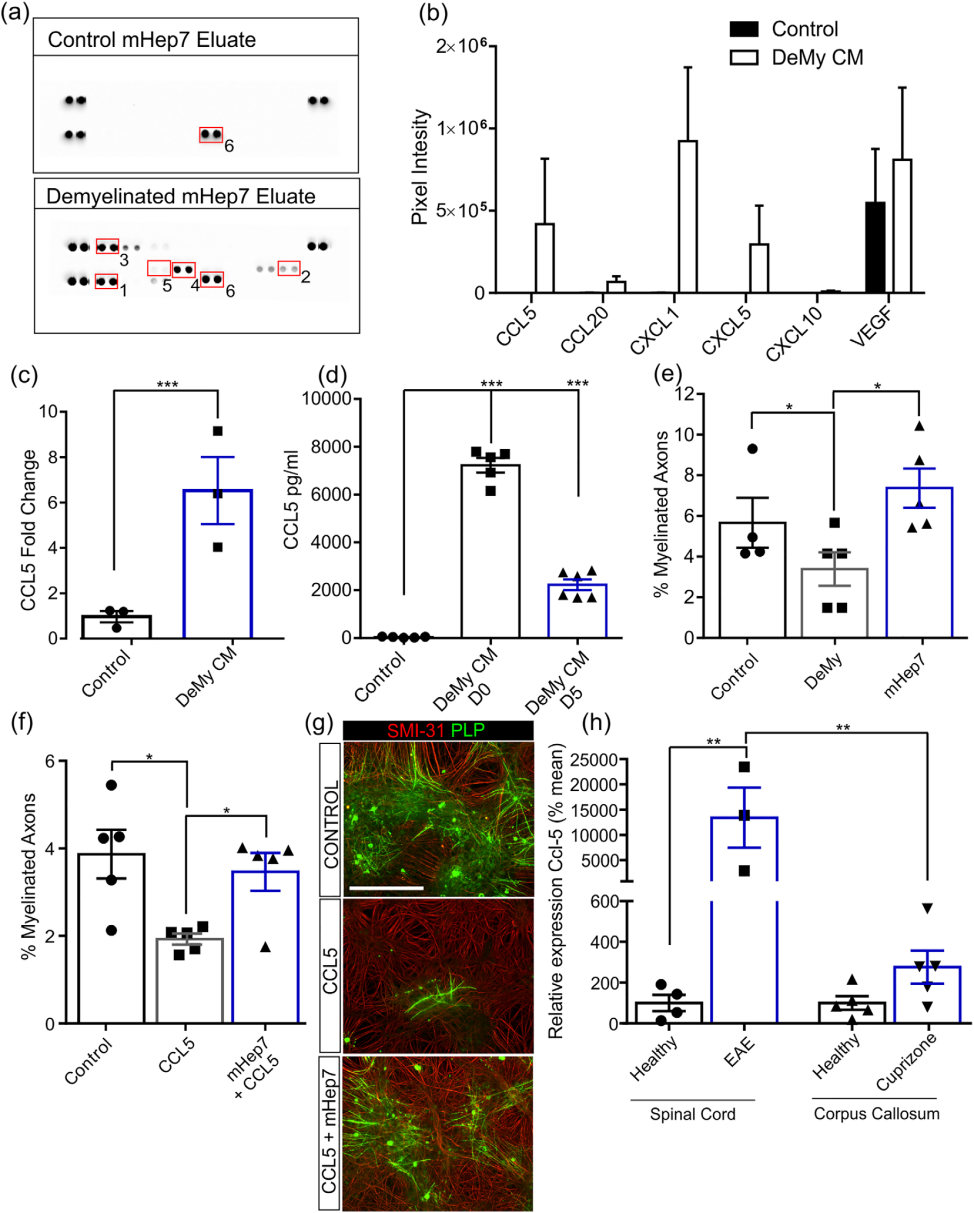
A potential role for CCL5 in LS‐mHep7 mechanism‐of‐action. (a) Control conditioned medium (Control) and demyelinated conditioned medium (DeMy CM) were collected from cultures on 25 DIV (1 day after demyelination). The conditioned media underwent LS‐mHep7 affinity chromatography, and the cytokine profile was assessed. Representative dot blots of the arrays for each conditioned media eluate are shown. (b) Quantification of dot blots based on pixel intensity. DeMy CM LS‐mHep7 eluate contained numerous factors not found in Control LS‐mHep7 eluate, including CCL5 (*n* = 3; technical replicates = 2). (c) Quantification of the fold change in pixel intensity showed a significant upregulation of CCL5 after demyelination (DeMy, ***p < .001, Student's *t* test). (d) CCL5 ELISA of conditioned media, collected from demyelinated cultures (DeMy) either immediately after demyelination (DeMy CM D0) or 5 days later (DeMy CM D5) confirmed a significant increase in CCL5 concentration compared with control cultures. (*n* = 3; technical replicates = 3). (e) LS‐mHep7 treatment (1 ng/mL) at 25 DIV (1 day after demyelination) promoted remyelination compared with untreated cultures. (f) CCL5 inhibits de novo myelination however, cotreatment with LS‐mHep7 (1 ng/mL) could overcome its inhibitory effect. (g) Representative images of control, CCL5 treated (100 ng/mL) or CCL5 and LS‐mHep7 treated myelinating cultures. Anti‐SMI‐31 stains axons in red, anti‐PLP stains myelin in green, Scale bar represent 100 μm. (h) Comparison of *Ccl‐5* expression in spinal cords of healthy versus EAE animals and the corpus callosum of healthy vs cuprizone‐induced animals. *Ccl‐5* was significantly upregulated in EAE spinal cords compared with healthy controls and was also upregulated compared with corpus callosum brain region of cuprizone fed animals. (Individual dots represent the experimental n number or individual animals in (h). One‐way ANOVA with Dunnett multiple comparison, or Two‐way ANOVA with Sikak's multiple comparison in (h) *p < .05. **p < .01, ***p < .001).

Chemokines and cytokines are known HS binding proteins that are important in EAE pathogenesis. They could therefore constitute a family of proteins through which LS‐mHep7 elicits its effect in vivo. To investigate potential candidates, an LS‐mHep7 affinity pull‐down was carried out on CM collected from in vitro CNS cultures which had been demyelinated (DeMy CM) and compared with CM collected from control cultures. The resultant eluates were assessed on a 29 plex array (Figure 5a). This screen identified several LS‐mHep7‐binding factors solely present in the CM following demyelination, including, CCL5, CCL20, CXCL1, CXCL5, and CXCL10 (Figure 5b). VEGF was expressed in both control and DeMy CM. CCL5 expression was sixfold greater than that produced by control cultures (Figure 5c). The increased upregulation of CCL5, both at 24 h and 5 days post‐demyelination compared with control cultures was confirmed by ELISA (Figure 5d). This suggests that demyelination triggers the enhanced secretion of CCL5 which is LS‐mHep7 binding. Interestingly, LS‐mHep7 treatment of DeMy cultures led to a recovery of myelination compared with control untreated cultures, implying that LS‐mHep7 can sequester negative factors released after demyelination, such as CCL5 (Figure 5e). We have previously shown that CCL5 is inhibitory to de novo myelination (Schultz et al., 2021), however when cultures are cotreated with LS‐mHep7, the negative effect of CCL5 is overcome (Figure 5f,g). To determine the potential contribution of CCL5 in each animal model, we next investigated the differential expression of *Ccl‐5* in EAE spinal cords, compared with the corpus callosum brain region in cuprizone fed animals (Figure 5h). *Ccl‐5* was substantially upregulated within EAE spinal cords 17 days postinduction compared with healthy spinal cord tissue. Contrary to this, after 3 weeks cuprizone ingestion followed by 17 days on normal chow, *Ccl‐5* was not upregulated to the same extent. In fact, *Ccl‐5* expression was 48.5‐fold greater in EAE spinal cords compared with the corpus callosum tissue of cuprizone intoxicated mice, suggesting that the amount of CCL5 present could be a contributing factor to the overall efficacy of LS‐mHep7. Collectively, these data suggest that the action of upregulated chemokines/cytokines that are expressed after demyelination, which are normally inhibitory to remyelination, can be abrogated by LS‐mHep7 treatment, supporting a mechanism‐of‐action that could underpin the ameliorating effects of LS‐mHep7 on disease progression in the EAE model.

## 4 DISCUSSION

Having shown the multitargeted therapeutic potential of heparin mimetics in vitro (Higginson et al., 2012; Lindsay et al., 2020; McCanney et al., 2019), here we provide the first evidence for efficacy of LS‐mHep7 in promoting in vivo recovery in two different animal models of demyelination; EAE and the cuprizone model. These complementary models induce oligodendrocyte death via differing mechanisms. EAE is an inflammatory immune‐mediated demyelinating model and a crucial tool for investigating potential preclinical treatments (Gold et al., 2006). Alternatively, cuprizone ingestion causes oligodendrocyte degeneration and apoptosis, microglial and astrocyte activation that induces demyelination in the corpus callosum, which spontaneously remyelinates over time upon cuprizone removal (Schmidt et al., 2013; Yakimov et al., 2019). These distinct models mean that we can assess the efficacy of LS‐mHep7 in demyelinating models in which the immune system has active involvement (EAE) versus one which involves a direct effect on glial cells (cuprizone).

### 4.1 LS‐mHep7 ameliorates EAE

LS‐mHep7 treatment produced a robust improvement in the EAE clinical disease course. Terminal EAE histology revealed that animals had significantly less lymphocytes recruited into the spinal cord inflammatory lesions. Although further studies are required to establish the mechanism of LS‐mHep7, it is likely that it modulates inflammation via its interaction with chemokine/cytokines and thus prevents the demyelination and axonal death seen in control animals. In addition, laminin upregulation, which is known to be increased in inflammatory lesions within EAE animals (Roscoe et al., 2009), was reduced with LS‐mHep7 treatment, potentially contributing to the reduced number of inflammatory cells crossing and damaging the BBB. Indeed, the speed in which LS‐mHep7 halted disease accumulation and immune cell infiltration after only two doses, suggests it likely interferes with or interrupts the receptor‐ligand interactions that occur during the initial autoreactive cascade. Animals at this early time point are not extensively demyelinated, suggesting it does not directly affect OPC differentiation or the process of remyelination per se, rather it halts the inflammatory cascade leading to demyelination and axonal pathology.

mHeps are regulators of multiple cellular mechanisms and are known to sequester factors secreted after injury that have a negative effect on repair (McCanney et al., 2019; Xu et al., 2018). In the EAE paradigm, LS‐mHep7 may sequester the large number of inflammatory cytokines and/or chemokines, which are drivers of EAE pathology. This complex multimodal action has been partially identified using Surfen, a small molecule aminoquinoline agent that binds and antagonizes both heparan sulfate proteoglycans (HSPGs) and chondroitin sulfate proteoglycans and has been tested as a treatment in EAE. With similar finding as this study, mice which received Surfen had lower numbers of lymphocytes in spinal cord lesions (Warford et al., 2018). Moreover, Surfen controlled T cell migration into the CNS by acting both at the periphery, as well as centrally. In particular, the reduced chemokine gradients of CCL2, CCL3 and CCL5 produced in Surfen treated animals were thought to be responsible for the lower number of inflammatory cells recruited. Interestingly, we also have shown that LS‐mHep7 binds CCL5 in vitro and sequesters its negative impact on myelination that we have reported previously (Schultz et al., 2021). However, despite Surfen reducing inflammatory responses, it inhibited remyelination (Warford et al., 2018), likely reflecting that this agent lacks specificity for HSPGs and has much broader effects through binding all sufated GAGs and potentially other anionic molecules. By contrast, LS‐mHep7, a monosulfated variant of natural heparin containing only *N*‐sulfation, is more HS specific, and anticipated to drive more selective and nuanced effects on HSPG functions, as observed through multiple studies with selectively desulfated heparins on modulation of biological processes (Duckworth et al., 2015; Higginson et al., 2012; McCanney et al., 2019; Patey et al., 2006; Scholefield et al., 2003).

It cannot be discounted that LS‐mHep7 also drives repair directly via promotion of OPC proliferation and differentiation within EAE spinal cord lesions since increased levels of MBP were found in terminal histological sections, or that it prevents axonal degeneration since there was also less axonal pathology. It is also possible that LS‐mHep7 blocks HS on the cell surface and/or in the ECM within inflammatory lesions, which could influence OPC function. Nonetheless, these differences are likely correlative to decreased animal score since EAE animals had less inflammatory cell infiltration at early timepoints, which are drivers of disease pathology. In addition, we have already shown that LS‐mHep7 has no effect on purified OPC proliferation or myelin development in complex in vitro CNS cocultures, suggesting they do not directly modulate OPC function (McCanney et al., 2019).

### 4.2 LS‐mHep7 in the cuprizone model

In the cuprizone model similar 40 mg/kg s.c. injections of LS‐mHep7 as used in the EAE model, did not improve remyelination compared with controls. Since the cuprizone model is due to the toxic death of oligodendrocytes (Praet et al., 2014), the lack of enhancement in remyelination could be linked to oligodendrocyte numbers. However, at the 2‐week time point investigated, there were no significant differences in OLIG2+ oligodendrocytes. In the cuprizone model, oligodendrocyte degeneration has been shown to be regulated by various factors including chemokines, such as CCL2 and CCL3 (Buschmann et al., 2012; Janssen et al., 2016), which are HS‐binding and could potentially be modulated by LS‐mHep7. In addition, it has been shown that HSPGs are fundamental to OPC differentiation (Properzi et al., 2008). In particular, syndecan, HS GAG synthesis and sulphation patterns change during oligodendrocyte development (Properzi et al., 2008). HS chains of different HSPG core proteins are differently sulfated in OPCs which is thought to be functionally important as it allows the binding of growth factors such FGF2, which interact with 2,6‐*O*‐sulfated residues (Properzi et al., 2008). Moreover, it has been shown that Sulf2 an extracellular sulfatase that can remove 6‐*O*‐sulfate from HS chains on OPCs and the ECM, which can indirectly influence OPC myelination via WNT and BMP signaling (Saraswat et al., 2021). However, we have previously shown in vitro using both purified OPCs and developmental MC, that LS‐mHep7 does not promote OPC proliferation or differentiation but stimulates their recruitment to the cut lesion edge (McCanney et al., 2019). This may be because LS‐mHep7 has been sulfated at the 2,6‐*O* position meaning it has less affinity for binding growth factors known to support OPC proliferation. It therefore appears that, in the cuprizone model, LS‐mHep treatment has no direct effect on OPCs proliferation or differentiation, although it cannot be discounted that analysis at an earlier time point in recovery may have revealed a different result. Furthermore, detailed analysis of mature oligodendrocytes or myelin ultrastructure using electron microscopy at earlier time‐points would perhaps also have yielded more conclusive results on the impact of LS‐mHep7. Thus, further studies would be needed to evaluate the possibility that LS‐mHep7 improves the rate of recovery in the cuprizone model.

### 4.3 LS‐mHep7 mechanism‐of‐action

It is clear from this investigation that s.c. injections of LS‐mHep7 ameliorated EAE disease but had no effect on remyelination within the cuprizone model. This could reflect the mechanistic differences of each animal model. EAE is driven by activation and expansion of CD4+ T‐lymphocytes (Billiau & Matthys, 2001), while the cuprizone model causes demyelination as a result of oligodendrocyte toxicity and death (Buschmann et al., 2012; Schmidt et al., 2013). The lack of any end‐point effect on remyelination in the cuprizone model could suggest that the predominant mechanism‐of‐action of LS‐mHep7 is through its modulation of the inflammatory response. It has been reported that heparin mimetics have antiinflammatory effects when used therapeutically in respiratory diseases such as asthma and chronic obstructive pulmonary disease (Mohamed & Coombe, 2017). A further difference is that the BBB remains largely intact in the cuprizone model (Kondo et al., 1987) but is disrupted during EAE (Lindsay et al., 2022). Therefore, it cannot be discounted that the greater access to the CNS environment in EAE mice may facilitate a more robust effect since we have shown that LS‐mHep7 can bind negative factors released after demyelination. Nevertheless, heparin‐derived oligosaccharides have already been shown to cross the BBB in in vitro cell culture models (Leveugle et al., 1998) and after intravenous or subcutaneous injection in vivo (Ma et al., 2002). In addition, we have been able to detect radiolabeled heparin‐mimetics with similar sulfate composition as LS‐mHep7 within healthy mouse brain tissue up to 24 h post oral gavage delivery (Turnbull Lab, unpublished observations), suggesting that this class of compound can effectively cross the intact BBB.

There could also be regional specificity (brain vs spinal cord) regarding the functional role of LS‐mHep7 in each model. For example, OPCs can exhibit different functional states that are influenced by their niche (Boshans et al., 2020). The tracts within the corpus callosum maintain plasticity throughout life, essential for the adaptive myelination required for new motor skills (Floriddia et al., 2020; McKenzie et al., 2014). This contrasts with the sensory afferent tracts within spinal cords which are likely more stable and require myelin favoring fast conduction (Floriddia et al., 2020). In addition, there could be spatial differences since it is known that after acute demyelination in the corpus callosum or the ventral funiculus of the spinal cord, OPCs from the dorsal region respond more robustly and make a greater contribution to remyelination than ventral derived OPCs (Crawford et al., 2016). It also cannot be excluded that gender‐related differences contributed to the disparity of LS‐mHep7 repair potential, since cuprizone intoxication was carried out using male C57BL/6 mice and the EAE model using female C57BL/6 mice. Yet, female, and male C57BL/6 mice have been shown to demyelinate and remyelinate similarly at the electron microscopic level in the cuprizone model, with similar levels of mature oligodendrocyte depletion and recovery (Taylor et al., 2010). Therefore, regardless of C57BL/6 mouse gender, the level of de/remyelination may have been the same in the cuprizone model, giving LS‐mHep7 a similar opportunity to stimulate repair. The impact of tissue niche or gender on the contribution to LS‐mHep7 efficacy requires further investigation.

### 4.4 LS‐mHep7 modulates weight gain and appetite

While general weight loss during EAE may be attributed to paralysis and reduced food intake, early weight loss prior to paralysis has been reported to be associated with the high production of pro‐inflammatory cytokines, such as TNFα, released during the acute phase of inflammation, causing a loss of appetite (Mardiguian et al., 2013; Tracey et al., 1988). Once the peak of EAE disease is reached, mice slowly gain weight, even if their clinical score does not improve. This increase in weight may be due to down‐regulation of inflammation, which results in lower levels of pro‐inflammatory cytokines in the blood. In this investigation, there was a prevention of disease‐associated weight loss and an overall increased weight of LS‐mHep7 treated EAE mice. While the exact mechanism requires further investigation, these data may provide additional evidence that LS‐mHep7 promotes appetite and weight gain via the dampening of the immune response. Animal weight has also been shown to be an important variable in the cuprizone model, since animals with a lower starting weight are more vulnerable to oligodendrocyte degradation and demyelination (Leopold et al., 2019). In the cuprizone model, which lacks an immunological component, LS‐mHep7 treatment did not cause animals to gain significantly more weight than time matched control animals, strengthening the suggestion that LS‐mHep7 main mechanism‐of‐action is via the dampening of the immune response. Overall, LS‐mHep7 may promote global physiological animal benefits in immune‐mediated demyelinating disease.

### 4.5 Role of CCL5 in LS‐mHep7 mechanism‐of‐action

To gain an insight into the mechanistic action of LS‐mHep7, the CM from demyelinated in vitro CNS cultures was analyzed. Analysis of the CM using a chemokine array showed that CCL5 was upregulated following demyelination, and importantly was LS‐mHep7 binding. The aberrant expression of chemokines and their receptors during MS has been well documented, including the upregulation of the chemokine CCL5 in MS lesions (Boven et al., 2000). Furthermore, polymorphisms modifying CCL5 and CCR5 genotypes in MS patients have been associated with clinical disease outcomes (van Veen et al., 2007). A recognized functional role of CCL5 in EAE is to regulate the recruitment of leukocytes (dos Santos et al., 2005; dos Santos et al., 2008). When anti‐CCL5 was administered following herpes virus‐induced demyelination, it attenuated T‐lymphocyte infiltration, thereby reducing demyelination severity (Glass et al., 2004). CCL5 treatment in vitro increases the proliferation of the oligodendroglial cell line Oli‐neu and induces a pro‐inflammatory response from microglia (Kadi et al., 2006; Skuljec et al., 2011). In this investigation, we established that CCL5 can inhibit myelination in vitro and that effect can be overcome by cotreatment with LS‐mHep7. Other research has shown that a trisulfated GAG mimetic can bind CCL5, consequently inducing the migration of CCR3 cells—CCR3 is a CCL5 receptor that plays an important role in chemotaxis (Sabroe et al., 2005; Sheng et al., 2013). This trisulfated GAG mimetic comprised two *O*‐sulfate moieties and a single *N*‐sulfate group. Interestingly, the LS‐mHep7 compound is only monosulfated containing only *N*‐sulfate groups; this implies that the *N*‐sulfate moiety may be sufficient for ligand binding. However, MC do not recapitulate leukocyte migration, hence the reduced myelination observed following CCL5 treatment, may be based on either the induction of a pro‐inflammatory environment by resident microglia or the promotion of OPC proliferation—which would in turn inhibit their differentiation into myelinating oligodendrocytes. Regardless of the mechanism, CCL5 is a negative factor for myelination that is known to be upregulated during EAE and MS.

CCL5 has also been implicated in pathogenesis of the cuprizone model. Its expression is increased 1 week after cuprizone ingestion, produced by resident microglia and astrocytes, although levels fall in the second and third week of diet ingestion (Biancotti et al., 2008; Zirngibl et al., 2022). CCL5 is considered a T cell‐specific protein, although it is produced by different cell types including monocytes/macrophages, microglia, astrocytes, and neurons (Rock et al., 2004). Therefore, in addition to being essential in leukocyte recruitment, CCL5 regulates microglia activation, survival, and migration to the site of injury (Skuljec et al., 2011). Therefore, it may play different roles in each model. In the EAE model, it aids leukocyte trafficking, but in the cuprizone model which lacks an immunological component it drives microglia activation. Microglia respond to myelin and oligodendrocyte debris following demyelination (Zirngibl et al., 2022) and they drive cuprizone‐induced demyelination (Marzan et al., 2021). Microglia numbers peak 3 to 5 weeks after cuprizone ingestion (Hiremath et al., 1998), but are rapidly cleared after cuprizone diet withdrawal (Lindner et al., 2009). In the cuprizone model, we commenced LS‐mHep7 treatment upon cuprizone diet withdrawal, when microglia numbers are known to have reduced. Correlating with this, we have shown that CCL5 expression in the corpus callosum following 3 weeks cuprizone intoxication is relatively low (not significantly different to healthy corpus callosum brain tissue) but remained substantially elevated in EAE spinal cords 17 days postinduction. Therefore, while CCL5 can be sequestered by LS‐mHep7, the lower levels present within the cuprizone model at the time of treatment may not facilitate repair.

Nevertheless, LS‐mHep7 is known to bind a plethora of chemokine, cytokines, and proteins and in this investigation, we identified not only CCL5, but CCL20, CXCL1, CXCL5, and CXCL10. In addition, we have identified 108 other proteins using TMT–LC/MS analysis of conditioned medium from demyelinated cultures, including amyloid beta A4, which we have shown to be inhibitory to myelination (McCanney et al., 2019). Therefore, it is highly unlikely that the therapeutic action of LS‐mHep7 is mediated via its binding of a single factor and more likely a result of it exerting biological effects through its modulation of numerous protein activity. These data suggest that LS‐mHep7 mechanistic action in EAE is mediated, at least partially, through the sequestering inflammatory chemokines that drive leukocyte trafficking into the spinal cord, one such example being CCL5.

The complex tissue damage seen after CNS injury/disease will require a multitarget approach, and our previous in vitro data supported the potential of LS‐mHeps as novel therapeutics since they regulate a diverse range of cellular functions (Lindsay et al., 2020; McCanney et al., 2019). In this investigation, we have confirmed for the first time the therapeutic potential of LS‐mHep7 in the EAE animal model of CNS injury, thus validating its candidature for future preclinical studies for the treatment of inflammatory autoimmune mediated disease.

## AUTHOR CONTRIBUTIONS

Project conceptualization Susan C. Barnett, Markus Kipp, and Jerry E. Turnbull; Designed and performed experiments Susan L. Lindsay, Zhan Jiangshan, Miriam Scheld and George A. McCanney; Methodology (synthesized LS‐mHep7) Edwin A. Yates; Designed experiments Susan C. Barnett, Susan L. Lindsay, Markus Kipp, and Jerry E. Turnbull; Formal analysis Susan L. Lindsay, Zhan Jiangshan, Miriam Scheld, Rebecca Sherrard Smith and George A. McCanney; Writing—original draft preparation Susan L. Lindsay and Susan C. Barnett; Writing—review and editing Markus Kipp, Jerry E. Turnbull, Edwin A. Yates, and Carl S. Goodyear; Resources (EAE project administration) Carl S. Goodyear; Funding acquisition Susan C. Barnett, Susan L. Lindsay, Markus Kipp, Edwin A. Yates, Jerry E. Turnbull.

## Supporting information


**FIGURE S1:** EAE and acute cuprizone model experimental timelines. (a) EAE was induced by s.c injection with 100 μL emulsion containing 150 μg MOG protein in CFA, followed by peritoneal injections of 200 ng of PTX on Day 0 and 2. Mice were treated from loss of tail tone and randomly divided into two groups: phosphate buffer saline (PBS) (*n* = 12) and LS‐mHep7 (*n* = 15). Animals were scored daily using the EAE clinical score. Animals were sacrificed at either an early time point 5 days post treatment or at the end of the experiment by terminal perfusion and stained using standard methods for IHC. (b) Four control animals received a diet of standard rodent chow for 5 weeks to determine normal levels of myelination (Control 1). Cuprizone intoxication was performed by the addition of 0.25% cuprizone in the standard rodent chow for 5 weeks and four animals were perfused to confirm demyelination (Control 2). After the 5 weeks, eight vehicle control animals received PBS (Group 1), eight animals were treated with LS‐mHep7 via s.c. (40 mg/kg; Group 2) every‐other‐day for 14 days. Animals were sacrificed at the end of the experiment by terminal perfusion and stained using standard methods for IHC.

**FIGURE S2:** Method of semiquantitative assessment of immunofluorescence positive staining within EAE spinal cord sections. (a) Images were taken of stained transverse thoracolumbar spinal cord sections. Specifically, regions with inflammatory lesions were analyzed. Example shows anti‐CD45 (green) and anti‐Laminin (red) (b) Two images per sections, with at least two sections per animal were analyzed for each marker of interest. The white matter region of interest (ROI) was selected manually in Image J using the freehand draw tool. (c) The regions outside the selected area were cleared leaving only the white matter ROI. Images were split into their different color channels and each thresholded using an in‐house developed pipeline. Thresholding values were kept consistent between animals stained with the same marker. (d). The entire white matter ROI was thresholded to obtain a pixel value for the entire area. (e) Quantification of staining was calculated by dividing the number of positive reactivity pixels by the number of white matter ROI pixels for each field of view which was then expressed as % threshold/area. Values were averaged across images obtained for each animal and presented as individual data points on the graph.

## Data Availability

Raw data were generated at the University of Glasgow. Derived data supporting the findings of this study are available from the corresponding author [SCB] upon reasonable request.
