# Supplementary figures and images for: Low sulfated heparan sulfate mimetic differentially affects repair in immune‐mediated and toxin‐induced experimental models of demyelination
Source: Glia. 2023 Mar 21;71(7):1683–98. doi: 10.1002/glia.24363 (PMC10952530; doi:10.1002/glia.24363)

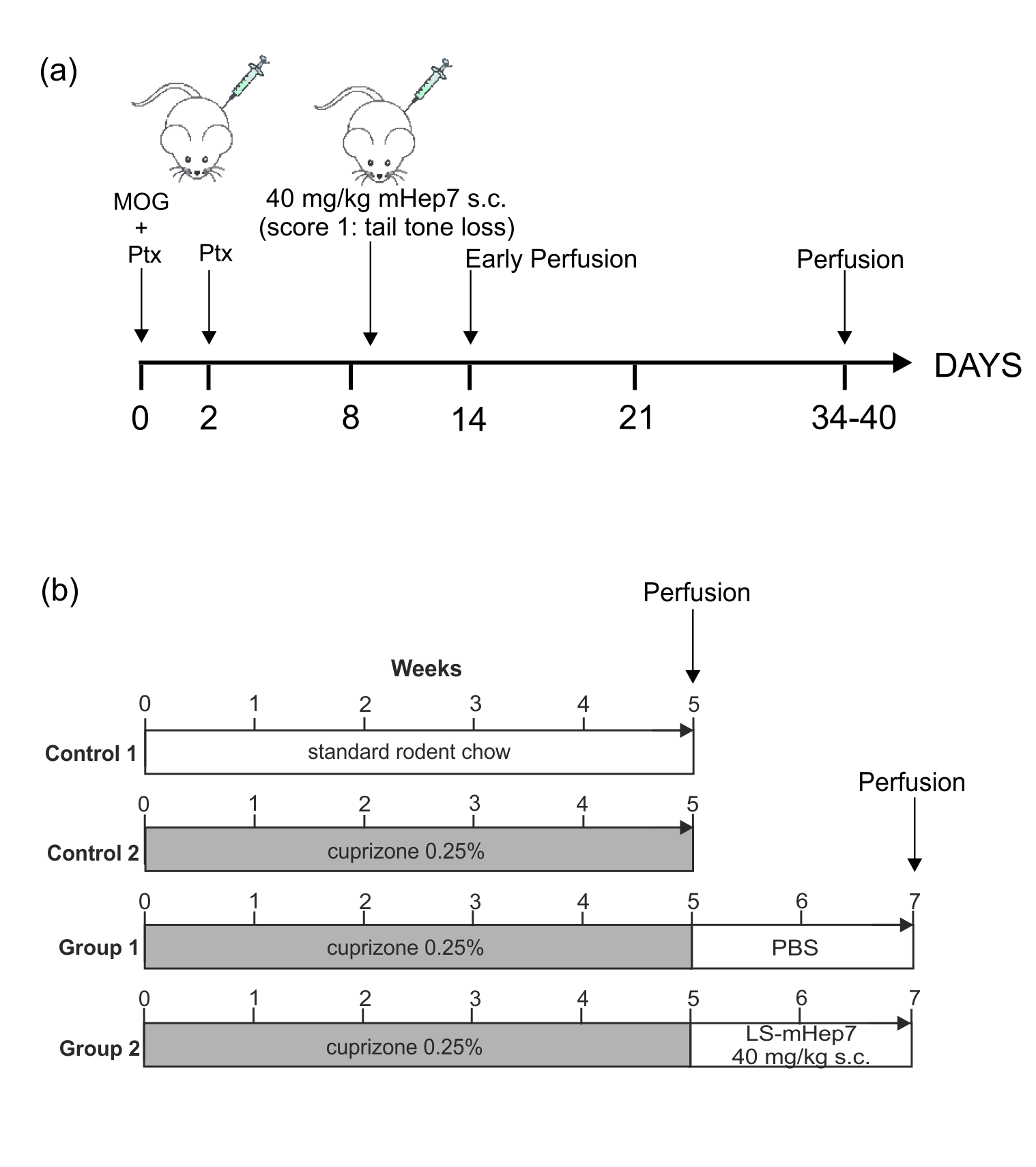

Supplement: Supplementary file 1 — FIGURE S1: EAE and acute cuprizone model experimental timelines. (a) EAE was induced by s.c injection with 100 μL emulsion containing 150 μg MOG protein in CFA, followed by peritoneal injections of 200 ng of PTX on Day 0 and 2. Mice were treated from loss of tail tone and randomly divided into two groups: phosphate buffer saline (PBS) (n = 12) and LS‐mHep7 (n = 15). Animals were scored daily using the EAE clinical score. Animals were sacrificed at either an early time point 5 days post treatment or at the end of the experiment by terminal perfusion and stained using standard methods for IHC. (b) Four control animals received a diet of standard rodent chow for 5 weeks to determine normal levels of myelination (Control 1). Cuprizone intoxication was performed by the addition of 0.25% cuprizone in the standard rodent chow for 5 weeks and four animals were perfused to confirm demyelination (Control 2). After the 5 weeks, eight vehicle control animals received PBS (Group 1), eight animals were treated with LS‐mHep7 via s.c. (40 mg/kg; Group 2) every‐other‐day for 14 days. Animals were sacrificed at the end of the experiment by terminal perfusion and stained using standard methods for IHC. [file GLIA-71-1683-s001.tif]

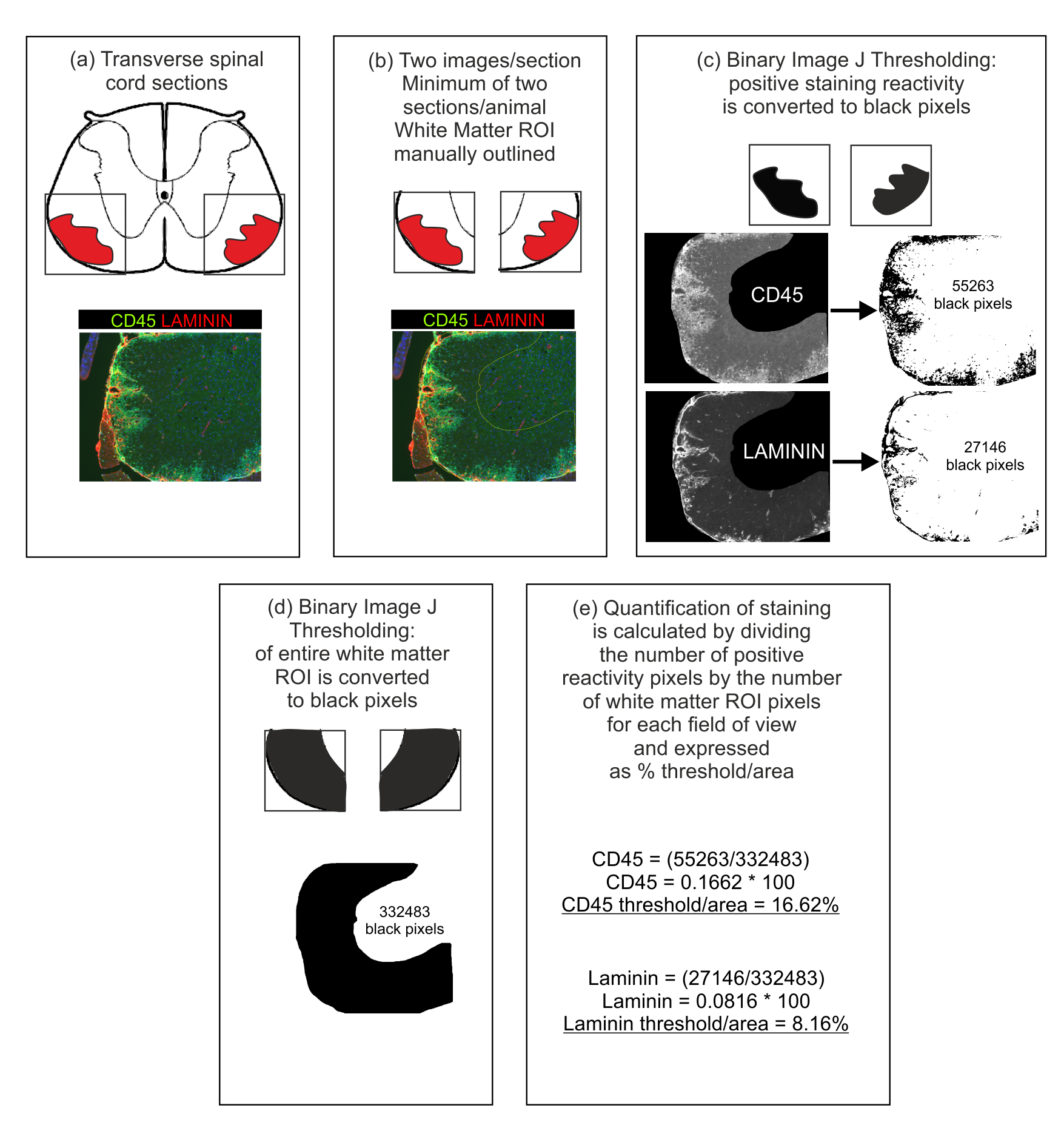

Supplement: Supplementary file 2 — FIGURE S2: Method of semiquantitative assessment of immunofluorescence positive staining within EAE spinal cord sections. (a) Images were taken of stained transverse thoracolumbar spinal cord sections. Specifically, regions with inflammatory lesions were analyzed. Example shows anti‐CD45 (green) and anti‐Laminin (red) (b) Two images per sections, with at least two sections per animal were analyzed for each marker of interest. The white matter region of interest (ROI) was selected manually in Image J using the freehand draw tool. (c) The regions outside the selected area were cleared leaving only the white matter ROI. Images were split into their different color channels and each thresholded using an in‐house developed pipeline. Thresholding values were kept consistent between animals stained with the same marker. (d). The entire white matter ROI was thresholded to obtain a pixel value for the entire area. (e) Quantification of staining was calculated by dividing the number of positive reactivity pixels by the number of white matter ROI pixels for each field of view which was then expressed as % threshold/area. Values were averaged across images obtained for each animal and presented as individual data points on the graph. [file GLIA-71-1683-s002.tif]
